# Supplementary figures and images for: Viral Entry Inhibitors Protect against SARS-CoV-2-Induced Neurite Shortening in Differentiated SH-SY5Y Cells
Source: Viruses. 2023 Sep 28;15(10):2020. doi: 10.3390/v15102020 (PMC10611151; doi:10.3390/v15102020)

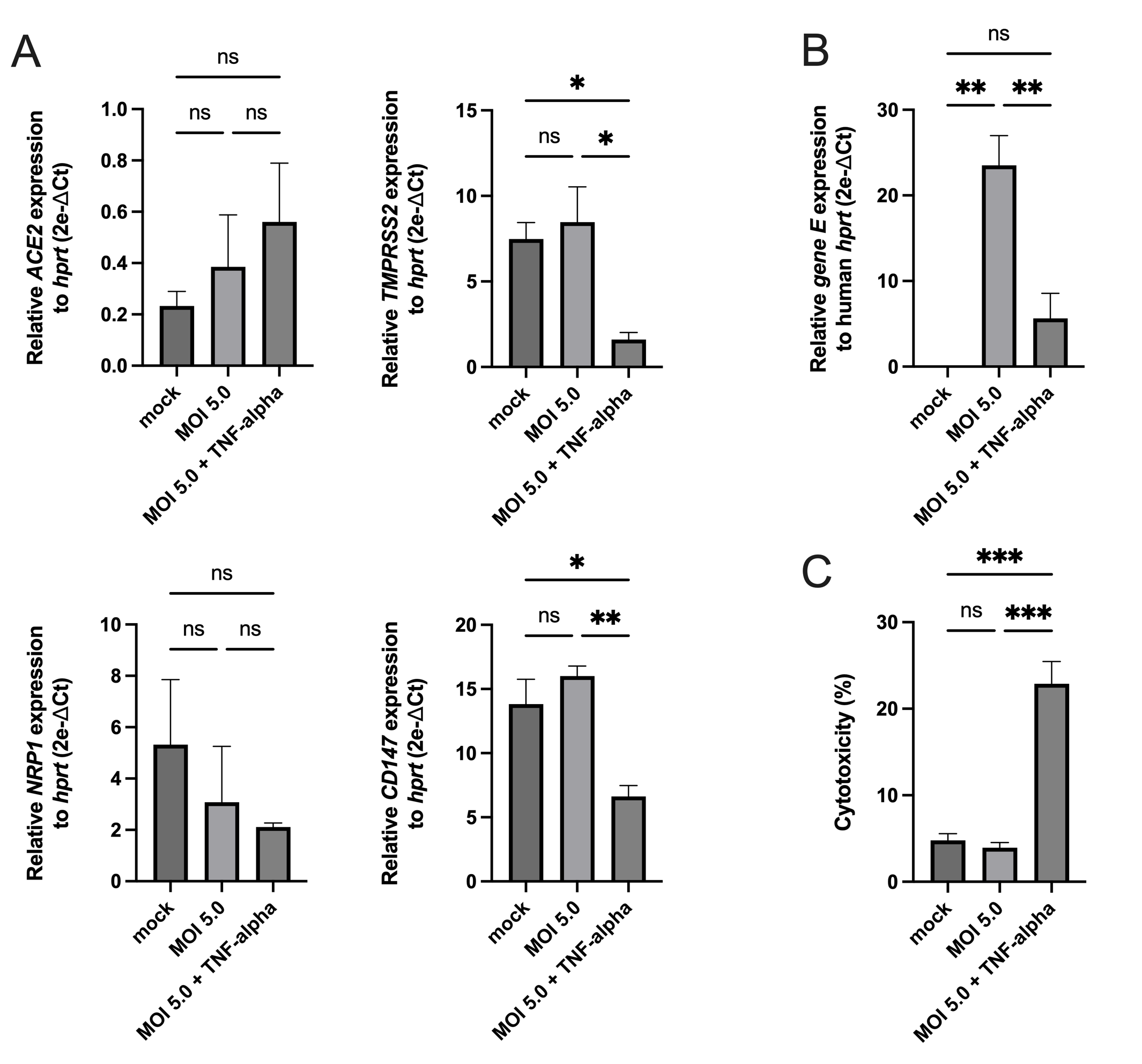

Supplement: Supplementary file 1 [file viruses-15-02020-s001.zip › FigureS1.tiff]

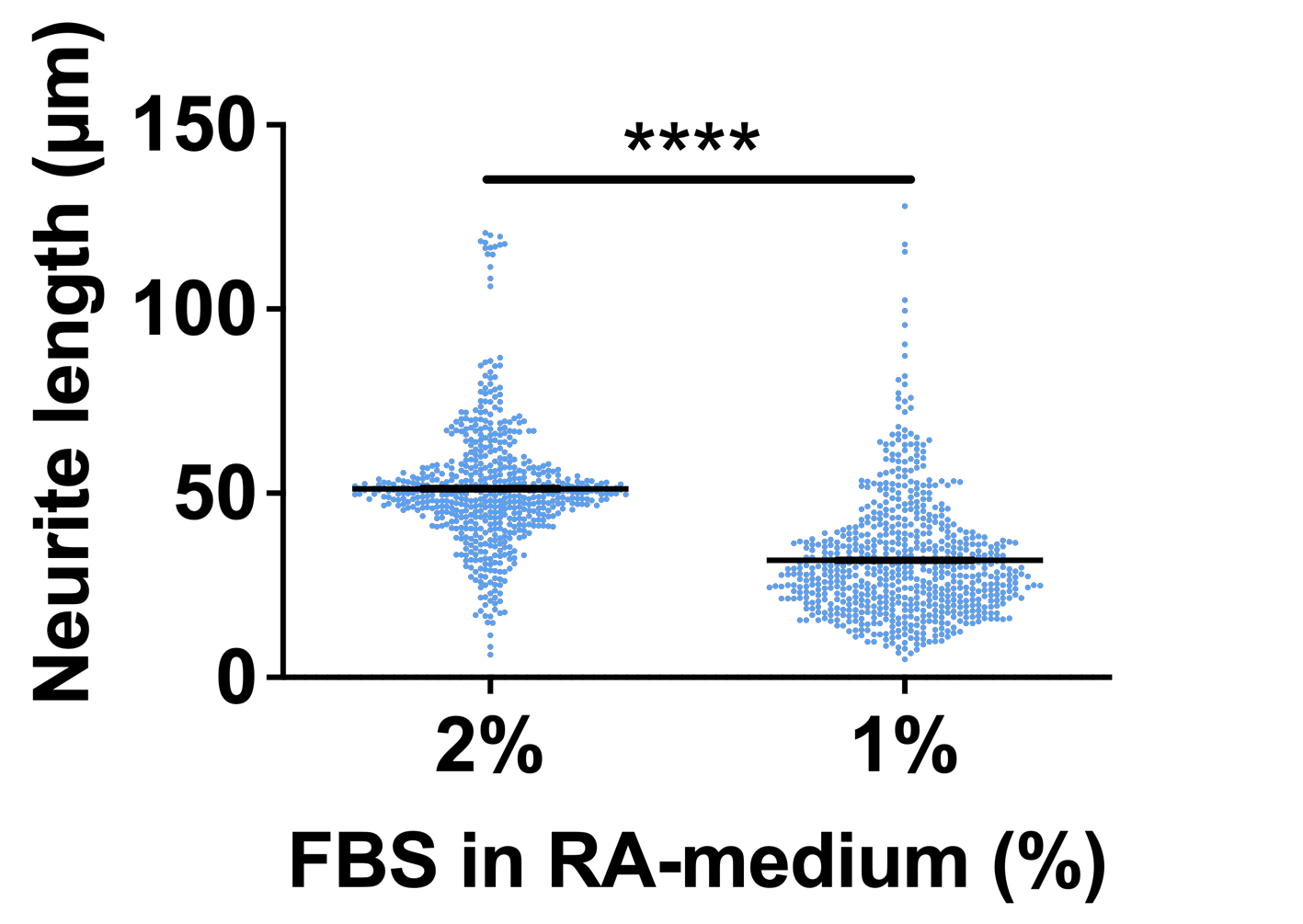

Supplement: Supplementary file 1 [file viruses-15-02020-s001.zip › FigureS2.tiff]
